# All-Trans Retinoic Acid Directs Urothelial Specification of Murine Embryonic Stem Cells via GATA4/6 Signaling Mechanisms

**DOI:** 10.1371/journal.pone.0011513

**Published:** 2010-07-13

**Authors:** Joshua R. Mauney, Aruna Ramachandran, Richard N. Yu, George Q. Daley, Rosalyn M. Adam, Carlos R. Estrada

**Affiliations:** 1 Urological Diseases Research Center, Children's Hospital Boston, Boston, Massachusetts, United States of America; 2 Department of Surgery, Harvard Medical School, Boston, Massachusetts, United States of America; 3 Department of Biological Chemistry and Molecular Pharmacology, Harvard Medical School, Boston, Massachusetts, United States of America; 4 Harvard Stem Cell Institute, Boston, Massachusetts, United States of America; 5 Stem Cell Transplantation Program, Division of Pediatric Hematology/Oncology, Children's Hospital Boston, Boston, Massachusetts, United States of America; 6 Dana Farber Cancer Institute, Boston, Massachusetts, United States of America; 7 Howard Hughes Medical Institute, Boston, Massachusetts, United States of America; 8 Division of Hematology, Brigham and Women's Hospital, Boston, Massachusetts, United States of America; 9 Manton Center for Orphan Disease Research, Boston, Massachusetts, United States of America; Cincinnati Children's Hospital Medical Center, United States of America

## Abstract

The urinary bladder and associated tract are lined by the urothelium, a transitional epithelium that acts as a specialized permeability barrier that protects the underlying tissue from urine via expression of a highly specific group of proteins known as the uroplakins (UP). To date, our understanding of the developmental processes responsible for urothelial differentiation has been hampered due to the lack of suitable models. In this study, we describe a novel in vitro cell culture system for derivation of urothelial cells from murine embryonic stem cells (ESCs) following cultivation on collagen matrices in the presence all trans retinoic acid (RA). Upon stimulation with micromolar concentrations of RA, ESCs significantly downregulated the pluripotency factor OCT-4 but markedly upregulated UP1A, UP1B, UP2, UP3A, and UP3B mRNA levels in comparison to naïve ESCs and spontaneously differentiating controls. Pan-UP protein expression was associated with both p63- and cytokeratin 20-positive cells in discrete aggregating populations of ESCs following 9 and 14 days of RA stimulation. Analysis of endodermal transcription factors such as GATA4 and GATA6 revealed significant upregulation and nuclear enrichment in RA-treated UP2-GFP+ populations. GATA4−/− and GATA6−/− transgenic ESC lines revealed substantial attenuation of RA-mediated UP expression in comparison to wild type controls. In addition, EMSA analysis revealed that RA treatment induced formation of transcriptional complexes containing GATA4/6 on both UP1B and UP2 promoter fragments containing putative GATA factor binding sites. Collectively, these data suggest that RA mediates ESC specification toward a urothelial lineage via GATA4/6–dependent processes.

## Introduction

Mechanisms regulating the development of epithelial tissues associated with the mammalian urogenital system are poorly understood. In particular, the sequential molecular cues necessary for the specification of the bladder urothelium have yet to be completely clarified. This transitional epithelium primarily serves as a highly selective permeability barrier protecting underlying tissues from toxic urinary components thereby preserving the integrity of the associated urinary tract and ultimately bladder and renal function [1]. Various congenital and acquired abnormalities including bladder and cloacal extrosphy [2], interstitial cystisis [3], neurogenic bladder secondary to myelomeningocele [4], and transitional cell carcinoma [5] are associated with aberrant urothelial differentiation and subsequent loss of normal barrier function. In order to devise novel therapies to address these conditions, an increased understanding of the regulatory networks involved in urothelial development is required.

The bladder urothelium is derived from the definitive endoderm [6], one of the three primary germ layers whose subsequent patterning and differentiation leads to the formation of a variety of major organs including the liver, pancreas, lungs, thyroid and intestines [7]. In the mouse, the definitive endoderm, together with mesoderm and ectoderm, is formed from the embryonic ectoderm of the epiblast through the process of gastrulation beginning at approximately day 6.5 of gestation [8]. The onset of bladder development begins with the formation of a transient embryonic cavity called the cloaca located at the caudal end of the hindgut, which is subsequently partitioned by the urorectal septum into a ventrally placed primitive urogenital sinus and dorsal anorectal canal by E8.0 [9]–[13].

Between E12–15, the urothelium develops from the urogenital sinus into a multi-layered epithelium composed of axially subdivided basal, intermediate, and superficial cell layers [14]. Basal cells represent a germinative zone which differentiates towards the lumen into a pre-maturation population defined as intermediate cells, and finally into fully differentiated superficial cells. Throughout the formation of the bladder urothelium, urothelial cells increase their expression of uroplakins (UP) which assemble into heterodimers and are essential for proper barrier function [15]–[18]. Genetic ablation of one or more uroplakin genes in mice causes severe retrograde vesicoureteral reflux, hydronephrosis, and renal failure, conditions that mirror certain human congenital diseases [1]. The ureter and trigone region of the bladder are also lined by a transitional urothelium thought to be derived from the mesoderm [19]. This is in contrast to the rest of the bladder and prostatic urethra which are patterned from the definitive endoderm, but these tissues are presumed to perform similar functions [20].

Vitamin A-mediated signaling pathways have been implicated in critical processes involved in the formation of the bladder from the urogenital sinus as well as the maintenance of a differentiated urothelial phenotype. Batourina and colleagues demonstrated that vitamin-A-rescued transgenic mice deficient in retinaldehyde dehydrogenase-2, an enzyme crucial for embryonic retinoic acid synthesis, exhibited a high rate of urogenital sinus abnormalities ranging in severity from bladder hypoplasia to almost complete bladder agenesis in which only a rudimentary urogenital sinus was formed [21]. These defects were found to be similar to malformations previously reported in vitamin A deficiency studies in rats [22]. Vitamin A deficiency can also induce keratinizing squamous metaplasia within the rodent bladder urothelium wherein the normal transitional epithelial structure becomes morphologically similar to the epidermis [23].

The major biologically active derivative of vitamin A, all trans retinoic acid (RA), is known to promote specification of a number of endodermal phenotypes during development including lung [24], kidney [25], intestine [26], and pancreas [27], however its role in urothelium formation is currently unknown. Specific target genes of RA signaling networks such as the GATA family of zinc finger transcription factors including GATA4/6 have been shown to participate in the maturation of extra-embryonic endoderm derivatives [28], [29] as well as cardiac [30] and bronchial epithelial [31] specification. In situ hybridization studies have demonstrated that GATA factors are also highly expressed in the urogenital ridge during bladder formation [32]. These results suggest that these proteins may be targets of retinoid signaling networks which contribute to urothelial lineage progression and ultimately play key roles in bladder specification and maturation.

In vitro models of bladder urothelial specification from embryonic precursors have the potential to generate novel cell sources for tissue engineering applications as well as serve as models of development. In the present study, we demonstrate the ability of all trans retinoic acid (RA) to induce lineage progression of murine embryonic stem cells (ESC) toward urothelial fate(s) in vitro. By interrogating this system, we established the role of specific GATA factors as essential downstream regulators of RA-mediated UP expression.

## Methods

### Cell Culture

Murine ESC (C57BL/6 line, American Type Culture Collection, Manassas, VA) were expanded on γ-irradiated mouse embryonic fibroblasts in Dulbecco's modified Eagle's medium (DMEM) supplemented with 15% fetal calf serum (FCS), 100 units/ml penicillin, 100 µg/ml streptomycin, 0.1 mM nonessential amino acids, 2 mM L-glutamine, 1% nucleosides, 0.1 µM 2-mercaptoethanol, and 10^3^ units/ml leukemia inhibitory factor (LIF) (expansion medium) and maintained in a humidified tissue culture incubator (37°C, 5% CO_2_). To induce cellular differentiation, feeder-depleted ESCs were seeded on 2D native type I collagen matrices [33] (50,000 cells/cm^2^) in DMEM supplemented with 15% FCS, 2 mM L-glutamine, 100 units/ml penicillin, 100 µg/ml streptomycin, 0.1 mM nonessential amino acids, 1 mM 1-thioglycerol, and all trans retinoic acid (RA) [0.1–10 µM] and cultured for up to 14 d with daily medium changes. Spontaneously differentiating controls in the absence of RA were maintained in parallel. In addition, transgenic ESC lines homozygous null (−/−) for GATA4 [34] or GATA6 [28] were also evaluated for their differentiation potential in response to RA stimulation.

### Construction of the mouse UP2 reporter plasmid and generation of UP2 stable ESC lines

A 3528 bp fragment of the mouse UP2 gene upstream of the transcription start site was generated by PCR amplification using Phusion high-fidelity DNA polymerase (New England Biolabs, Ipswich, MA) and a mouse UP2 specific primer set (forward: 5′-CGTCTCGAGGATCTCGGCCCTCTTT-3′; reverse: 5′-GGACTGGATCCTGGAACAGGTG-3′). The resulting PCR fragment was subcloned into the pCR4Blunt vector (Invitrogen, Carlsbad, CA). DNA sequencing of isolated plasmid clones was performed on an automated DNA analyzer (Model 3730, Applied Biosystems, Foster City, CA). The UP2 promoter was then subcloned into the ClaI + BamHI sites of the pGreenZeo-SR500VA reporter vector (System Biosciences, Mountain View, CA). The resulting plasmid, pSR500-mUP2, was further modified to express G418 resistance. A KpnI/XhoI cassette isolated from the pPNT plasmid [35] containing the neomycin resistance gene under the control of the constitutively active PGK promoter was transferred into the Kpn I + Sal I sites of pSR500-mUP2 to create the pSR500-NEO-mUP2 plasmid. The pSR500-NEO-mUP2 plasmid was introduced into ESCs by Amaxa nucleofection (Lonza, Basel, Switzerland) and the cells were plated and expanded onto G418-resistant MEFs (Applied StemCell, Sunnyvale, CA) in expansion medium detailed above. Twenty-four h after nucleofection, G418 (Invitrogen) was added to the medium at a final concentration of 200 µg/ml and selection was continued for 8 d. Individual clones were isolated and further expanded on MEF cells in expansion medium supplemented with 20 µg/ml G418 in order to maintain transgene integration. Clones were subjected to differentiation analysis detailed above.

### mRNA Analysis

Total RNA was extracted from ESC cultures according to the single step acid-phenol guanidinium method [36] using Trizol reagent (Invitrogen, Carlsbad, CA). mRNA was enriched from total RNA using the RNeasy kit (Qiagen Inc., Valencia, CA) according to the manufacturer's instructions. cDNA were synthesized using High-Capacity cDNA Transcription kit (Applied Biosystems, Foster City, CA) following the manufacturer's instructions. Real time RT-PCR reactions were performed and analyzed using the ABI Prism® 7900HT Sequence Detection System (Applied Biosystems) and SDS Plate Utility Software (version 2.1). cDNA samples were assessed for genes of interest and the housekeeping gene, GAPDH, in independent reactions using the Taqman Universal PCR master mix in combination with commercially available primers and probes consisting of Assays-on-Demand™ Gene Expression kits (Applied Biosystems) following the manufacturer's instructions. Expression kits included: UP1A, Mm01176597_g1; UP1B, Mm00769504_m1 UP2, Mm00447665_m1; UP3A, Mm00447665_m1, UP3B, Mm00558406_m1, GAPDH, Mm99999915_g1; OCT-4, Mm00658129_gH; cytokeratin 1, Mm00492992_g1; cytokeratin 10, Mm03009921_m1; cytokeratin 18, Mm01601706_g1; cytokeratin 20, Mm00508106_m1; GATA4, Mm03053570_s1; GATA5: Mm00484692_m1; GATA6, Mm00802636_m1; SOX7, Mm00776876_m1; SOX17, Mm00488363_m1; FOXA1, Mm00484713_m1; FOXA2, Mm00839704_mH; α-fetoprotein, Mm00431715_m1; PEM, Mm00476718_m1; p63, Mm00495788_m1; CXCR4, Mm01292123_m1; HOXA13, Mm00433967_m1; smooth muscle myosin heavy chain (SM-MHC), Mm00443013_m1; nestin, Mm03053244_s1; PDX1, Mm00435565_m1; and Nkx3.1 Mm00440479_m1. For each cDNA sample, the threshold cycle (Ct) was defined as the cycle number at which amplification of the target gene was within the linear range of the reaction. Relative expression levels for each gene of interest were calculated by normalizing the target gene transcript level (Ct) to the respective GAPDH level as described previously (2^−ΔΔCt^ formula, Perkin Elmer User Bulletin #2).

### Immunocytochemistry and Immunohistochemistry

Cells were fixed with neutral buffered formalin and UP, p63, CK20, α-actin, and GATA4/6 expression was detected by immunofluorescence using the following primary antibodies: anti-pan-uroplakin [rabbit antisera raised against total bovine uroplakin extracts, TT Sun from New York University, 1∶100 dilution], anti-p63 [Santa Cruz Biotechnology, Santa Cruz, CA, anti mouse, 1∶200 dilution], anti-CK20 [Santa Cruz Biotechnology, anti-rabbit, 1∶200 dilution], anti-α-actin [Sigma-Aldrich, anti-mouse, 1∶200] anti-GATA4 [Santa Cruz Biotechnology, anti-rabbit, 1∶200] and GATA6 [Santa Cruz Biotechnology, anti-rabbit, 1∶200] followed by incubation with species-matched Cy3, FITC, or Texas Red-conjugated secondary antibodies. For experiments demonstrating co-localization of UP2-GFP+ cells with GATA4/6 expression, GFP expression was enhanced utilizing an anti-GFP primary antibody (Abcam, Cambridge, MA, anti-mouse, 1∶200) followed by detection with a FITC-conjugated secondary antibody. Nuclei were counterstained with 4′, 6-diamidino-2-phenylindole (DAPI), and specimens were visualized using an Axioplan2 fluorescence microscope (Carl Zeiss MicroImaging, Thornwood, NY). In addition, adult C57BL/6 mice were euthanized according to IACUC-approved protocols and urinary bladders were isolated, fixed in neutral-buffered formalin for 2 h, dehydrated in graded alcohols, paraffin-embedded and cut into 5 µm thick sections. Sections were analyzed for urothelial and epithelial markers as described above.

### Flow cytometry

Following 14 d of culture, RA-stimulated and spontaneously differentiating UP2-GFP ESCs were dissociated into a single cell suspension and quantified for GFP fluorescence on a FACScaliber flow cytometer (BD Biosciences, Franklin Lakes, NJ). Untransfected ESCs either maintained as controls or differentiated similarly were analyzed in parallel in order to control for background fluorescence. CellQuest 3.0 software (BD Biosciences) was used to acquire and analyze FACScan data. In some experiments, GFP+ populations were sorted and collected from RA-treated UP2-GFP ESCs utilizing a MoFlo high-speed sorter (Dako-Cytomation, Carpinteria, CA) and subjected to further analysis detailed below.

### Protein Analysis

Whole cell lysates were retrieved in 20 mM Tris-Cl, pH 7.4, 150 mM NaCl, 1 mM EDTA, 1 mM EGTA, and 1% Triton X-100 supplemented with protease and phosphatase inhibitors. Nuclear extracts were obtained according to methods reported in the literature [37]. Immunoblot analysis was performed as described previously [38]. Primary antibodies included: anti-p63 and GATA4/6 as described above.

### Electromobility shift analysis

Electromobility shift analyses (EMSAs) for GATA-4 and GATA-6 binding to murine uroplakins 1B and 2 promoter sequences were carried out as using methods previously described [39]. Briefly, 3–5 µg of nuclear extract and 0.5 ng of ^32^P-labeled oligonucleotide probe were combined in a reaction mix (20 µl reaction volume) containing 25 mM Tris-HCl, pH 8, 50 mM KCl, 5 mM MgCl_2_, 0.5 mM EDTA, 8% glycerol, 2 µg of BSA, and 0.5 µg of poly(dI-dC). The reaction was incubated for 20 min at room temperature, and the DNA-protein complexes were resolved on 5% polyacrylamide gels in 0.5X Tris-borate-EDTA (TBE) buffer at 4°C. The gels were dried, and the complexes were visualized by autoradiography using a Typhoon Trio variable mode phosphorimager (GE Healthcare Biosciences, Piscataway, NJ). For competition experiments, a 25-fold molar excess of the cold competitor oligonucleotide was added simultaneously with the probe. For supershift experiments, a GATA4 or GATA6 antibody (as described above) was preincubated on ice for 1 h with nuclear extract followed by addition of the other components for 20 min at room temperature. Species-matched IgG antibodies were used in parallel as additional controls.

The following oligonucleotides were used in EMSAs: UP1B Gata wt, corresponding to putative GATA binding motif present in 2 kb UP1B promoter fragment (5′-GCC TTT GGG GAGATA GCA CTA ATC TAT-3′); UP1B Gata mut1, containing a point mutation (underlined) in putative GATA binding motif present in 2 kb UP1B promoter fragment (5′- GCC TTT GGG GA GGTA GCA CTA ATC TAT -3′); UP1B Gata mut2, containing a dual point mutation (underlined) in putative GATA binding motif present in 2 kb UP1B promoter fragment (5′- GCC TTT GGG GA CTTA GCA CTA ATC TAT -3′); UP2 Gata wt, corresponding to putative GATA binding motif present in 2 kb UP2 promoter fragment (5′- GTG TGC ATG TGG ATA AGT GTG TGT GTG -3′); UP2 Gata mut1, containing a point mutation (underlined) in putative GATA binding motif present in 2 kb UP2 promoter fragment (5′- GTG TGC ATG TGG GTA AGT GTG TGT GTG -3′); UP2 Gata mut2, containing a dual point mutation (underlined) in putative GATA binding motif present in 2 kb UP2 promoter fragment (5′- GTG TGC ATG TGC TTA AGT GTG TGT GTG -3′).

### Statistical Analysis

All quantitative measurements were collected with N = 3–4 independent determinations per data point and expressed as mean ± standard deviation. Data for these measurements were analyzed with Microsoft Excel software utilizing a Student's two-tailed t-test assuming equal levels of variance. Statistically significant values were defined as *p*<0.05.

### Ethics Statement

All animals were handled in strict accordance with good animal practice as defined by the standards outlined in the National Research Council's Guide and Children's Hospital Boston (CHB) PHS Assurance, and all animal work was approved by the CHB Animal Care and Use Committee (IACUC, Protocol 08-09-1207).

## Results

### Effect of RA on ESC UP Expression

**Figure 1:**
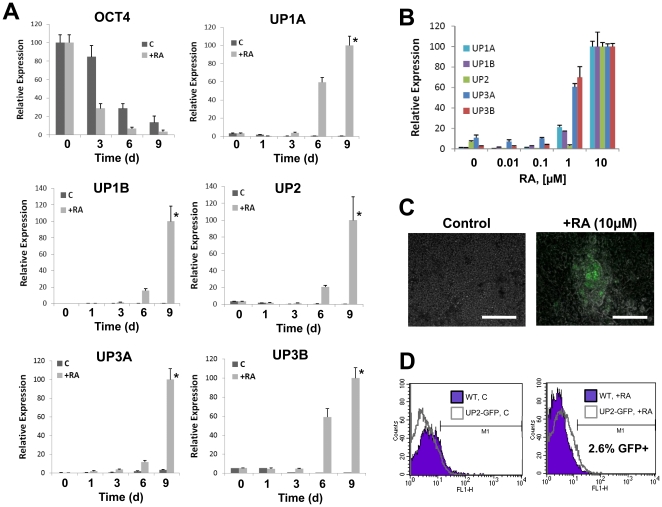
RA stimulation of ESCs induces UP expression in a time and concentration-dependent manner. [**A**] Real time RT-PCR analysis of UP and OCT-4 expression by ESCs cultured on collagen matrices in the presence (+RA) or absence (C) of 10 µM RA for up to 9 d. (*) = p<0.05, in comparison to spontaneously differentiating controls and undifferentiated ESCs. [**B**] Effect of RA concentration on UP expression following 9 d. For [**A–B**], levels normalized to GAPDH expression. Mean ± SD per data point. [**C**] Photomicrographs of merged phase and GFP expression in spontaneously differentiating controls and RA-stimulated UP2-GFP ESC line following 14 d of cultivation. Scale bar = 80 µm. [**D**] Flow cytometry histograms comparing the extent of GFP expression in untransfected (WT) and UP2-GFP ESC lines either as spontaneously differentiating controls (left panel) or RA-treated cultures following 14 d (right panel). Extent of fluorescence intensity per amount of population events is displayed with shaded plots representing WT cultures and open plots representing UP2-GFP ESC line.

RA is known to function in both a time- and concentration-dependent manner to selectively regulate lineage specification during ESC differentiation [27], [40], [41]. We first investigated the ability of continuous cultivation of ESCs in the presence of RA (0.01–10 µM) to stimulate urothelial marker expression. Real time RT-PCR analysis demonstrated that withdrawal of LIF as well as the addition of micromolar concentrations of RA led to the down-regulation of OCT-4 mRNA transcript levels in ESC cultures, indicating a progression toward differentiation (**Figure 1A**). In addition, RA acted synergistically with LIF withdrawal to promote pluripotency marker decline. RA stimulation increased mRNA transcript levels of all five uroplakins (UPs) over those seen in spontaneously differentiating controls and naïve ESCs by day 6 of cultivation with significant elevation occurring by 9 d and continuing to rise through 14 d of culture (**Figure S1**). The ability of RA to stimulate UP expression was concentration-dependent, with micromolar concentrations stimulating maximal extents of mRNA transcription over the 9 d time course (**Figure 1B**).

Following 14 days of cultivation, RA stimulation (10 µM) of a stable UP2-GFP ESC line led to substantial upregulation of GFP fluorescence in discrete cell populations primarily localized within 3-D cellular aggregates dispersed throughout the culture (**Figure 1C**). In addition, flow cytometry revealed approximately 2.6±0.5% of the RA-stimulated population was GFP+ compared to WT cultures maintained in parallel (**Figure 1D**). Spontaneously differentiating controls and naïve ESCs demonstrated negligible GFP fluorescence as assessed by fluroscence microscopy and flow cytometry analysis. Real time RT-PCR analysis of endogenous UP2 mRNA transcript levels in sorted GFP+ cell populations derived from RA-treated cultures demonstrated substantial elevation of expression in comparison to unfractionated populations and GFP- fractions (**Figure S2**). Together, these results demonstrate that GFP expression is RA-responsive and correlates with endogenous UP2 transcription.

### UP expression is associated with lineage markers associated with the stratified urothelium of the urogenital tract

**Figure 2:**
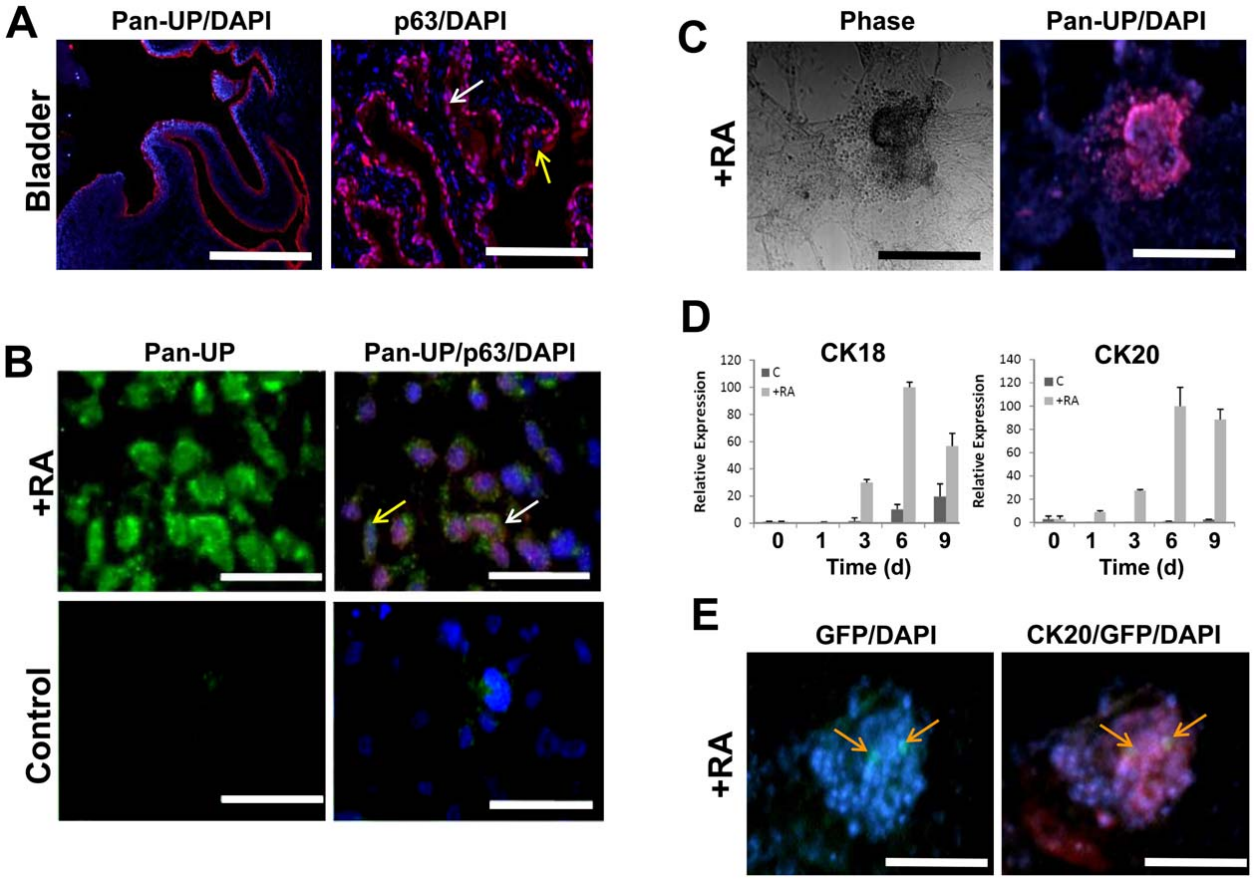
RA enrichment of UP+ populations coincides with markers of stratified urothelium. [**A**] Photomicrographs of adult murine bladder (C57BL/6) showing native urothelium organization and architecture. Left panel: immunofluorescence of pan-UP expression (red, Cy3) localized in urothelium. Scale bar = 500 µm. Right panel: immunofluorescence of nuclear p63 expression (red, Cy3) localized to the basal and intermediate urothelial layers (denoted with white arrow). Absence of nuclear p63 staining was noted in superficial urothelial cells (denoted with yellow arrow). Scale bar = 100 µm. For both panels, images were merged with DAPI nuclear counterstain (blue). [**B**] Photomicrographs of spontaneously differentiating control and RA-treated ESCs following 9 d of cultivation showing acquisition of epithelial morphology and co-localization of UP (green, FITC) and nuclear p63 expression (red, Cy3). Pan-UP+ populations merged with DAPI were observed with (denoted with white arrow) and without (denoted with yellow arrow) p63+ nuclear staining. Scale bar = 30 µm. [**C**] Photomicrographs of phase and immunofluorescence fields of RA-treated ESCs following 14 d of cultivation. Samples demonstrated 3-D cell aggregates coupled with pan-UP expression (red, Cy3). DAPI nuclear counterstain (blue). Scale bar = 100 µm. [**D**] Real time RT-PCR analysis of CK18 and CK20 expression in cultures described in [**B**]. Levels normalized to GAPDH expression. Mean ± SD per data point. [**E**] Photomicrographs of immunofluorescence fields of RA-treated UP2-GFP ESCs following 14 d of cultivation demonstrated GFP expression colocalized with CK20 expression (red, Cy3). Cells exhibiting co-staining of GFP and Cy3 are denoted with orange arrows. DAPI nuclear counterstain (blue). Scale bar = 60 µm.

IHC analysis of UP protein expression in the adult murine bladder demonstrated specific localization throughout each layer of the urothelium with maximal expression observed in the apical layer of the superficial umbrella cells lining the lumen (**Figure 2A**). Previous studies have reported the expression of UPs and their assembly into AUM is essential for the maintenance of urothelial barrier function [15]–[18]. Transgenic mice lacking the basal cell marker p63 present severe epithelial defects, including epidermis and prostate bud agenesis and loss of basal and intermediate urothelial cell layers [42]. Our results demonstrated nuclear p63 expression localized to the urothelium of the murine bladder with detection restricted to basal and intermediate cell layers. These data were consistent with previous observations of urothelial marker distribution in the murine bladder [15]–[16], [42].

UP protein expression was first detected in RA-treated cultures following 9 d of cultivation and was localized to 2-D cell nests distributed throughout the culture population (**Figure 2B**). Further ICC analysis demonstrated the presence of both p63+ and p63- cell fractions within the UP+ population (**Figure 2B**). Immunoblotting demonstrated enrichment of p63 expression in nuclear fractions of RA-treated cultures compared to spontaneously differentiating controls (**Figure S3**). By 14 d of RA stimulation, UP expression was further detected in selective 3-D cell aggregates and peripheral populations (**Figure 2C**). The distribution of UP protein was similar to that observed within UP2-GFP+ subpopulations identified by promoter-reporter assays detailed above.

Murine urothelium is also characterized by a unique pattern of cytokeratin (CK) expression. It has been confirmed that among cytokeratins, CK20 expression is restricted to the superficial cells of normal adult urothelium [14], while CK18 is expressed in all three cell layers during development, but diminishes postnatally with maturation [14]. Real time RT-PCR analysis of CK18 and CK20 expression in RA-treated ESCs revealed significant upregulation of mRNA transcript levels over controls (**Figure 2D**). However, differential expression kinetics were detected for these markers wherein CK20 plateaued during the onset of UP expression (6–9 d) while CK18 peaked following 6 d and declined by 9 d of treatment. Similar analysis of cytokeratins associated with vaginal (CK1) and keratinizing (CK10) epithelium [43], demonstrated negligible induction over controls following 9 d of RA treatment (data not shown). In addition, ICC revealed robust CK20 protein expression associated with selective UP2-GFP+ populations within the 3-D cell aggregates present in RA-stimulated cultures (**Figure 2E**).

### Selective RA concentrations stimulate expression of early endoderm transcription factors

**Figure 3:**
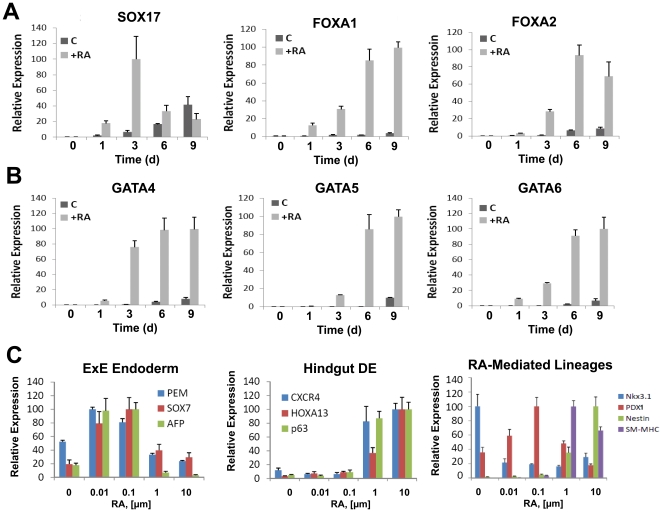
RA induction of UP expression is correlated with markers of hindgut definitive endoderm (DE), in contrast to markers of the extraembryonic endoderm (ExE). [**A, B**] Real time RT-PCR analysis of endoderm marker expression by ESCs cultured on collagen matrices in the presence (+RA) or absence (C) of 10 µM RA for up to 9 d. [**C**] Effect of RA concentration on ESC differentiation markers of ExE or hindgut DE, as well as various RA-responsive lineages following 9 d of stimulation. For all panels, levels were normalized to GAPDH expression. N = 3–4 per data point.

RA is known to promote differentiation of murine ESCs toward particular endodermal phenotypes via stimulation of transcriptional networks that mediate embryonic patterning in vivo. In order to evaluate potential pathways and transcriptional mediators responsible for urothelial specification, we first assessed the temporal expression of early endoderm transcription factors, SOX17 [44] and its associated downstream targets, FOXA1 [45] and FOXA2 [46] (**Figure 3A**), as well as GATA 4/5/6 (**Figure 3B**) in relation to the onset (6–9 d) of RA-mediated UP expression.

Real time RT-PCR analysis demonstrated significant upregulation of SOX17, GATA4 and GATA6 as early as d 1 following RA stimulation. Kinetic analysis revealed that SOX17 peaked at 3 d of RA stimulation and receded to control levels by 9 d. Maximal upregulation of FOXA1 and FOXA2 was noted at 6–9 d, presumably as a consequence of increased SOX17 expression. GATA4 mRNA transcript levels peaked in response to RA at d 3 and plateaued until d 9. ESCs also increased levels of GATA5 and GATA6 expression, both of which peaked at 9 d of continuous stimulation with RA. These results demonstrate that RA-mediated upregulation of SOX17, its targets FOXA1 and FOXA2, GATA4/5/6 occurs in a temporal fashion coinciding with the onset and progression of urothelial marker expression in ESCs.

### Selective RA concentrations stimulate specification of various types of endoderm and associated derivatives

UP expression is primarily restricted to the urothelial compartment of the urogenital tract. However, several reports have also described expression of selected UPs at divergent sites throughout the developing mammalian embryo including UP1B within the intestines, ocular epithelium, and the tissues of the extra-embryonic endoderm such as the allantois [47]. Early endodermal markers such as SOX17 and GATA factors have also been identified as crucial signaling molecules in directing both extraembryonic endoderm (ExE) and definitive endoderm development [28], [34], [44], [48]. Therefore expression of these markers is not mutually exclusive for lineage discrimination. To further understand the origin of urothelial marker expression in ESC cultures, we investigated the impact of various RA concentration regimes over a 9 d time course on ExE marker expression and assessed this trend relative to UP induction (**Figure 3C**). Real time RT-PCR analysis demonstrated substantial upregulation of ExE markers including the global ExE transcription factor, PEM [49], SOX7 (parietal endoderm) [50], and α-fetoprotein (visceral endoderm) [51] in response to 0.01–0.1 µM of RA over non-stimulated controls, while 10 µM RA resulted in no significant upregulation over control levels. These results demonstrate that RA modulates progression toward ExE lineages within a concentration range which is 100-fold less than the optimal concentration responsible for induction of UP expression in vitro.

Since the bladder urothelium is derived from the definitive hindgut endoderm following its specification from the urogenital sinus, we further evaluated the effect of RA concentration on modulation of markers selective for the development of hindgut endoderm derivatives and compared this trend to the onset of UP induction (**Figure 3C**). CXCR4 has been proposed as a marker of definitive endoderm [52] and the presence of this protein within the urinary bladder has been implicated as an important signaling molecule in mediating normal micturition [53]. The homeobox (Hox) family of transcription factors also represents crucial signaling molecules that regulate embryonic patterning and organogenesis [54]. Studies from *Hoxa-13*−*/*− transgenic mice, have shown the loss of this transcription factor results in a hypoplastic urogenital sinus with an absence of the presumptive bladder anlage [55]. Analysis of RA-treated ESC populations demonstrated significant upregulation of CXCR4 and Hoxa13 mRNAs, together with p63 expression in response to µM concentration regimes. Temporal progression of marker expression corresponded with the induction of urothelial markers and plateaued with similar kinetics (**Figure S4**).

### UP expression is associated with specific types of RA-stimulated lineages

Since RA-treated cultures were observed to consist of morphologically heterogeneous populations, we investigated the presence of other RA-responsive and/or endodermal-derived lineages (**Figure 3C**). In parallel with increased UP expression, RA in the µM range was sufficient to induce maximal expression of SM-MHC and nestin, over nonstimulated controls. These changes are indicative of both SMC and neuronal lineages, respectively, and are consistent with previous reports [40], [56]. Optimal expression of other definitive endoderm lineage markers including PDX1, representative of pancreatic differentiation [27], was confined to 0.1 µM RA, while Nkx3.1, a marker of prostate epithelium [57], another hindgut derivative, was strongly down-regulated compared to controls. Markers of skeletal muscle, Myf5 [58], and hematopoietic lineages, GATA1 [59], in cultured ESCs were unresponsive to micromolar concentrations of RA (data not shown), while the lung epithelial marker, surfactant protein C [60], was undetectable by real time RT-PCR analysis. These results suggest that micromolar concentrations of RA are selective for definitive endoderm progression as opposed to ExE endoderm, while the former is enriched for the urothelial lineage, in contrast to certain other gut derivatives. Markers specific for mesoderm and ectoderm derivatives which populate the bladder such as SMCs and neurons were induced in parallel with urothelial differentiation; these divergent lineages may be regulated by a general RA-mediated mechanism responsible for bladder specification.

### Role of GATA factors in RA-mediated stimulation of UP expression

**Figure 4:**
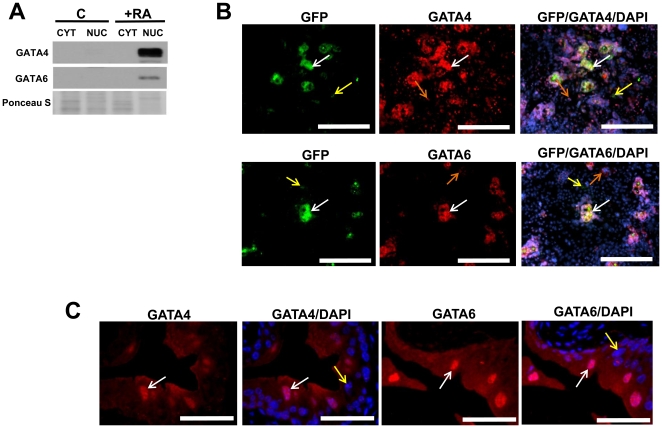
Nuclear GATA4 and GATA6 expression is associated with UP expression both in vitro and in vivo. [**A**] Immunoblot analysis of nuclear (NUC) and cytoplasmic (CYT) protein fractions demonstrating enrichment of GATA4 and GATA6 in nuclear extracts of wild type (WT) RA-treated ESCs following 9 d of cultivation. The degree of Ponceau S staining of membranes after transfer was used to indicate uniform sample loading. [**B**] Photomicrographs of RA-treated UP2-GFP+ cells co-stained for GFP (green, FITC) and nuclear GATA4/6 (red, Cy3) following 14 d of culture. Observed populations included: GFP/GATA4/6+ cells (denoted by white arrows), cells positive for GATA4/6 expression alone (denoted by orange arrows), and GFP+, but GATA4/6 negative cell types (denoted by yellow arrows). Scale bar = 500 µm. [**C**] Photomicrographs of adult murine (C57BL/6) bladder urothelium showing nuclear localization of GATA4/6 in superficial cells (denoted by white arrows), while exclusion in the basal and intermediate cell layers (denoted by yellow arrows). Scale bar = 40 µm. For both [**A, B**], images were merged with DAPI nuclear counterstain (blue).

Mechanisms responsible for RA stimulation of UP expression are unknown. However, our results demonstrate that mRNA transcript levels of certain GATA factors are upregulated in parallel during this process. Immunoblot analysis demonstrated enrichment of GATA4 and GATA6 in nuclear fractions of RA-treated ESCs over control levels following 9 d of cultivation (**Figure 4A**). Nuclear localization of GATA4 and GATA6 was also evident by ICC analysis in various morphologically distinct RA-stimulated subpopulations at 6, 9, (**Figure S5A**) and 14 d of culture (**Figure 4B**). Moreover, expression of these GATA factors was co-localized to certain UP2-GFP+ subpopulations following 14 d of RA stimulation (**Figure 4B**). IHC staining of murine bladder urothelium further demonstrated robust nuclear expression of both GATA4 and GATA6 in superficial umbrella cells which strongly co-expressed UPs as detected using a pan-uroplakin antibody (**Figure 4C** **).** These results demonstrate that both in vitro and in vivo expression of UP is associated with the presence of nuclear GATA4/6.

Similar degrees of GATA4 and GATA6 expression were also evident in GFP- cell types at 14 d of culture. ICC analysis revealed that particular GFP- subpopulations not only co-expressed these GATA factors, but also displayed robust α-actin expression, a prominent smooth muscle-associated marker [39] (**Figure S5B**). These results are consistent with other published reports demonstrating expression of GATA4 and GATA6 in various smooth muscle phenotypes [39], [61].

**Figure 5:**
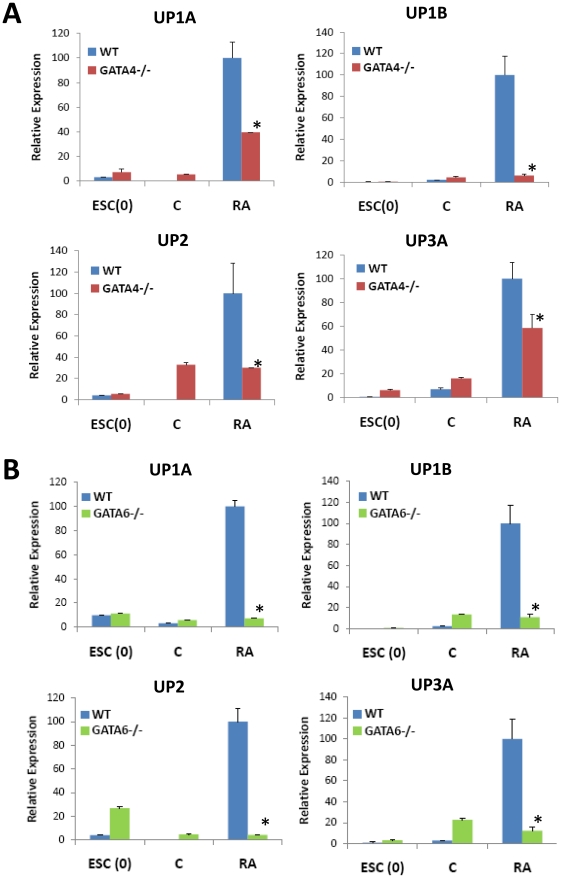
GATA4 and GATA6 are crucial signaling molecules in RA-mediated upregulation of UP expression in ESCs. Real time RT-PCR analysis of uroplakin expression in WT, GATA4−/− [**A**] or GATA6−/− [**B**] ESCs cultured in the presence (+RA) or absence (C) of 10 µM RA for up to 9 d. ESC(0)  =  undifferentiated ESCs. Levels normalized to GAPDH expression. Mean ± SD per data point. N = 3–4 per data point. (*) = p<0.05, in comparison to levels observed with RA-treated WT cultures.

Previous reports have demonstrated that knockout mice deficient in either GATA4 or 6 are embryonic lethal at E6.5–8, prior to the onset of bladder specification due to deficiencies in extraembryonic tissue formation [28], [34]. To determine the role of GATA4/6 in the regulation of markers associated with urothelial differentiation, GATA4−/− and GATA6−/− homozygous null ESCs were subjected to RA stimulation (10 µM) for 9 d and compared to wild type (WT) controls. Real time RT-PCR analysis revealed that mutant ESC lines were capable of expressing low baseline levels of certain UPs similar to WT controls. However, RA-treated GATA4−/− ESCs failed to enhance UP1B and UP2 expression in response to RA treatment as compared to unstimulated controls (**Figure 5A**). In addition, UP1A and UP3A expression were significantly attenuated compared to wild type cultures stimulated with RA in parallel. GATA6−/− ESCs failed to upregulate mRNA transcript levels of any of the major UPs (**Figure 5B**). These results demonstrate that GATA4/6 transcription factors play essential roles in regulating UP expression in RA-stimulated ESCs.

### Murine UP 1B and 2 promoters contain GATA factor binding sites which recruit GATA4 and GATA6 in response to RA stimulation

Our observations provide evidence that (1) RA-stimulated UP mRNA transcript levels are attenuated by the loss of GATA4/6 DNA binding activities and (2) RA stimulation is capable of enriching certain GATA factors in nuclear fractions of UP2-GFP+ cells (3) murine superficial urothelial cells are capable of co-expressing GATA4/6 as well as UP. These findings suggest that GATA 4/6 may be recruited to the promoters of various uroplakins, thus exerting positive transcriptional control over gene expression. Previous studies by Lin and colleagues demonstrated that a 3.6-kb 5′-flanking sequence of the mouse UP2 promoter was sufficient to promote transgene expression exclusively in the suprabasal cell layers of the urothelium, in a pattern similar to that of the endogenous UPII gene. These results provide evidence that many of the cis elements that define the bladder specificity and differentiation dependence within the UP2 gene reside in this 3.6-kb sequence [62].

**Figure 6:**
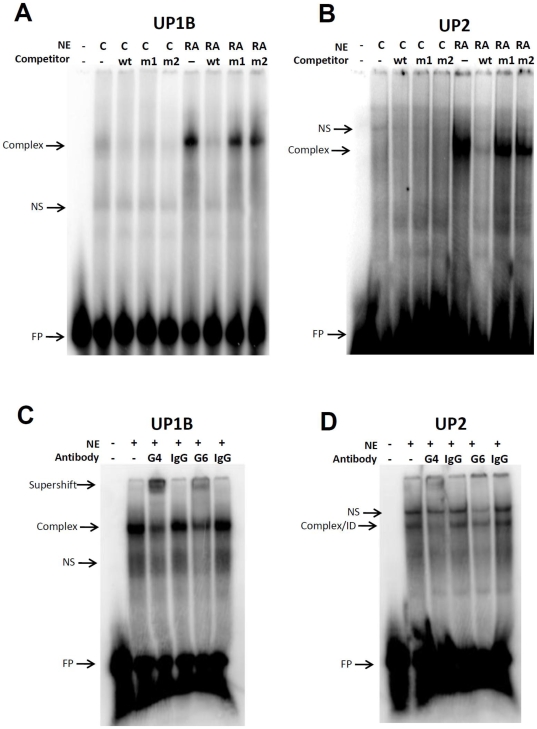
Murine UP1B and UP2 promoters contain GATA-DNA binding sites which recruit complexes containing GATA4/6 in response to RA stimulation. Nuclear extracts from RA-treated ESCs following 9 d show distinct GATA-DNA complexes with ^32^P-labeled consensus oligos specific for GATA binding to 2 kb UP1B [**A, C**] or UP2 [**B, D**] promoter fragments. Complexes were absent in spontaneously differentiating controls (C). Both complexes were inhibited by addition of respective excess unlabeled wild type (wt) but not mutant GATA oligos (m1, m2). [**C, D**] The presence of GATA4 (G4) and GATA6 (G6) in both the GATA-UP complexes was determined by supershift or immunodepletion (ID) following antibody-specific incubation (G4, G6), but not species-matched isotype control antibodies (IgG). NS represents non specific band. FP denotes excess free probe.

To determine the presence of putative GATA binding elements within the UP promoters, we analyzed a 2.0 kb fragment upstream from the transcriptional start site of each murine UP promoter sequence in silico using commercially available MatInspector software (Genomatix, Ann Arbor, MI) as previously described [63]. We identified one putative GATA-binding site within both the UP1B and UP2 promoter sequences at the following positions relative to the transcriptional start site: UP1B (−698 to −710) and UP2 (−1872 to −1885). In addition, promoter regions of the UP1A, UP3A, UP3B genes contained multiple putative GATA-binding sites. To determine whether GATA4/6 were recruited to the UP1B and UP2 promoters, we performed EMSA using nuclear extracts of RA-treated ESCs and spontaneously differentiating controls. As shown in **Figure 6A and 6B**, we observed robust complex formation with labeled probes corresponding to the GATA binding sites in both the UP1B and UP2 promoter sequences of RA-treated samples, while control nuclear extracts showed negligible binding. The complexes were competed out with the corresponding unlabeled UP1B or UP2 consensus GATA oligonucleotides, but not with mutant versions of these sequences. In addition, we observed supershift of the UP1B complex in the presence of GATA4 and GATA6 antibodies (**Figure 6C**), consistent with the presence of GATA4/6 in the complex. Supershift was also noted with the UP2 complex in the presence of GATA4 antibody, but not with IgG control; however the presence of GATA6 antibody resulted in substantial immunodepletion of the UP2 complex (**Figure 6D**) relative to IgG control. Together, these data suggest that RA-mediated signals promote recruitment of GATA4/6 to GATA binding sites within the UP1B/2 promoters during ESC specification.

## Discussion

Previous studies have provided some evidence that ESCs harbor urothelial differentiation potential in vitro and in vivo. Oottamasathien and colleagues reported that ESCs combined with fetal embryonic mesenchyme were able to form bladder-like structures expressing UP following 42 d of incubation within a kidney capsule model [64]. A study by Kinebuchi and colleagues showed cultivation of ESCs on collagen type I matrices in selective keratinocyte medium promoted upregulation of UP2 mRNA, however subcutaneous implantation was needed in order to detect UP protein expression [65]. The results of the present study further establish the potential of murine ESC to acquire molecular and biochemical features associated with urothelial phenotypes.

Our in vitro model is distinct from other systems since RA promotes expression of all UPs, together with developmental markers of the definitive endoderm in both a temporal and concentration-dependent manner in vitro. Our results indicate that UP-expressing cells generated using this model may originate specifically from a hindgut definitive endodermal population, in contrast to lineages of the ExE endoderm, which are also known to express certain UPs during development. In response to RA stimulation, lineage analysis of markers of various hindgut derivatives such as the prostate epithelium demonstrated significant down-regulation of Nkx3.1 in comparison to control levels. These results therefore suggest that UP-expressing populations observed within our system are unlikely to be associated with this lineage. In addition, the absence of CK1 upregulation in RA-treated cultures provides evidence that UP expression was not derived from vaginal epithelial differentiation.

Localization of UP expression with markers such as p63, CK20, and GATA4/6 in organized 3-D cellular aggregates provides evidence that RA stimulation may initiate various phenotypes associated with stratified urothelium. The presence of ESC-derived populations co-expressing nuclear p63 in combination with UPs suggests the presence of basal and intermediate urothelial lineages, given that similar trends in immunostaining were observed within the bladder urothelium in vivo. Our observation that some UP2-GFP+ populations express CK20 and GATA4/6 may also be evidence of superficial cell formation, since this cell type displays similar features in vivo [14]. Our observation that GATA4/6 nuclear localization occurs within the urothelium represents the first account to our knowledge of the presence of these transcription factors within this tissue compartment. In addition, the inability of RA treatment to upregulate CK10 in our culture system is also consistent with a lack of keratinizing epithelium which is predominant in the epidermis as well as in squamous metaplasia of the urothelium [43]. In addition, the decline in CK18 expression in RA-treated cultures coupled with the onset of UP expression at d 6 of culture may also be an indication of the maturation of urothelial populations, given the transient expression of this marker in vivo during bladder urothelial development and loss during postnatal maturation [14].

Collectively, our data suggest that in vitro UP-expressing populations resemble stratified epithelium from the urogenital tract. However, since the urothelium of both the bladder and ureter share many of the same morphological features and perform similar functions [43], we were unable to discriminate between these two possible lineages in this study. Previous urothelial differentiation models deploying ESCs as well as the results from this study have also demonstrated co-differentiation of both smooth muscle and neuronal lineages in an RA-responsive manner. These results may suggest a general RA-mediated mechanism which governs specification of urogenital-associated phenotypes from pluripotent stem cell sources.

The ability of RA to stimulate GATA4/6 mRNA and protein expression, complex formation at GATA-DNA binding sites within the UP1B and UP2 promoter sequences, and UP induction in ESCs suggests mechanism(s) by which retinoids modulate progression of certain urogenital epithelial lineages through particular GATA transcription factors. Retinoids are known to primarily facilitate changes in gene expression through binding to two classes of nuclear receptors, retinoic acid receptors (RARs) and retinoid X receptors (RXRs), which belong to the nuclear hormone receptor superfamily of transcription factors [66]. At least three genes encode each type of receptor (denoted α, β, and γ), and typical responses are transduced through binding of homo- and hetero-dimers of RAR and RXR receptor complexes to retinoic acid response elements (RAREs) present in the promoters of target genes [67]. Although, it is unknown how RA signals stimulate GATA4/6 expression in this system, previous studies of pluripotent F9 teratocarcinoma cells have identified GATA4 and GATA6 as transcriptional targets of RARγ [68] and RARβ2 [69] signaling complexes, respectively. These results coupled with observations from this study may suggest the existence of a signaling network in murine ESCs wherein selective RAR nuclear receptor complexes including RARγ and RARβ2 induce GATA4 and GATA6 expression leading to UP upregulation.

In summary, we described a novel protocol for the urothelial specification of murine ESCs and implicate RA-mediated GATA4/6 signaling mechanisms in the regulation of UP expression. The ability of our differentiation protocol to generate cell populations exhibiting multiple features of the urothelium is a potential advantage over other reported systems which require various periods of in vivo implantation to achieve comparable results. This feature may allow for isolation and expansion of clinically relevant populations which could be deployed in urogenital tissue engineering applications. Future investigations will focus on interrogating the utility of these ESC-derived populations to both integrate and contribute to urothelial function in vivo.

## Supporting Information

Figure S1 RA stimulation of ESCs further induces uroplakin expression from 9 to 14 days of culture. Real time RT-PCR analysis of UP expression by ESCs cultured on collagen matrices in the presence (+RA) or absence (C) of 10 µM RA from 9 to 14 d of culture. Levels normalized to GAPDH expression. Mean ± SD per data point.
(0.44 MB TIF)

Figure S2 Endogenous uroplakin 2 expression of FACS-sorted UP2-GFP ESC lines in response to RA stimulation. Real-time RT-PCR analysis of endogenous UP2 expression in UP2-GFP ESC lines cultured for 14 d in the presence of RA. Samples consisted of either unfractionated controls, GFP positive and negative fractions. Levels normalized to GAPDH expression. Mean ± SD per data point.
(0.42 MB TIF)

Figure S3 Enrichment of p63 in nuclear fractions of RA-treated ESCs. Immunoblot analysis of nuclear (NUC) and cytoplasmic (CYT) fractions demonstrating enrichment of p63 in nuclear extracts of wild type (WT) RA-treated ESCs following 9 d of cultivation.
(0.49 MB TIF)

Figure S4 RA stimulation of ESCs promotes upregulation of hindgut definitive endoderm markers in a time dependent manner. Real time RT-PCR analysis of definitive hindgut endoderm markers in response to RA stimulation (10 µM) over 9 d of cultivation. Undifferentiated and spontaneously differentiating controls (C) were analyzed in parallel. Levels normalized to GAPDH expression. Mean ± SD per data point.
(0.44 MB TIF)

Figure S5 RA stimulation of ESCs promotes temporal expression of nuclear GATA4/6 in various subpopulations including SMC phenotypes. [A] Nuclear GATA4/6 expression in RA-treated ESCs following 6 and 9 d of culture. Scale bar = 60 µm. [B] Photomicrographs of RA-treated ESCs co-stained for α-actin (green, FITC) and nuclear GATA4/6 (red, Cy3) (denoted by white arrows) following 14 d of culture. Scale bar = 500 µm. For both [A, B], images were merged with DAPI nuclear counterstain (blue).
(9.41 MB TIF)
