# Supplementary figures and images for: All-Trans Retinoic Acid Directs Urothelial Specification of Murine Embryonic Stem Cells via GATA4/6 Signaling Mechanisms
Source: PLoS One. 2010 Jul 13;5(7):e11513. doi: 10.1371/journal.pone.0011513 (PMC2903484; doi:10.1371/journal.pone.0011513)

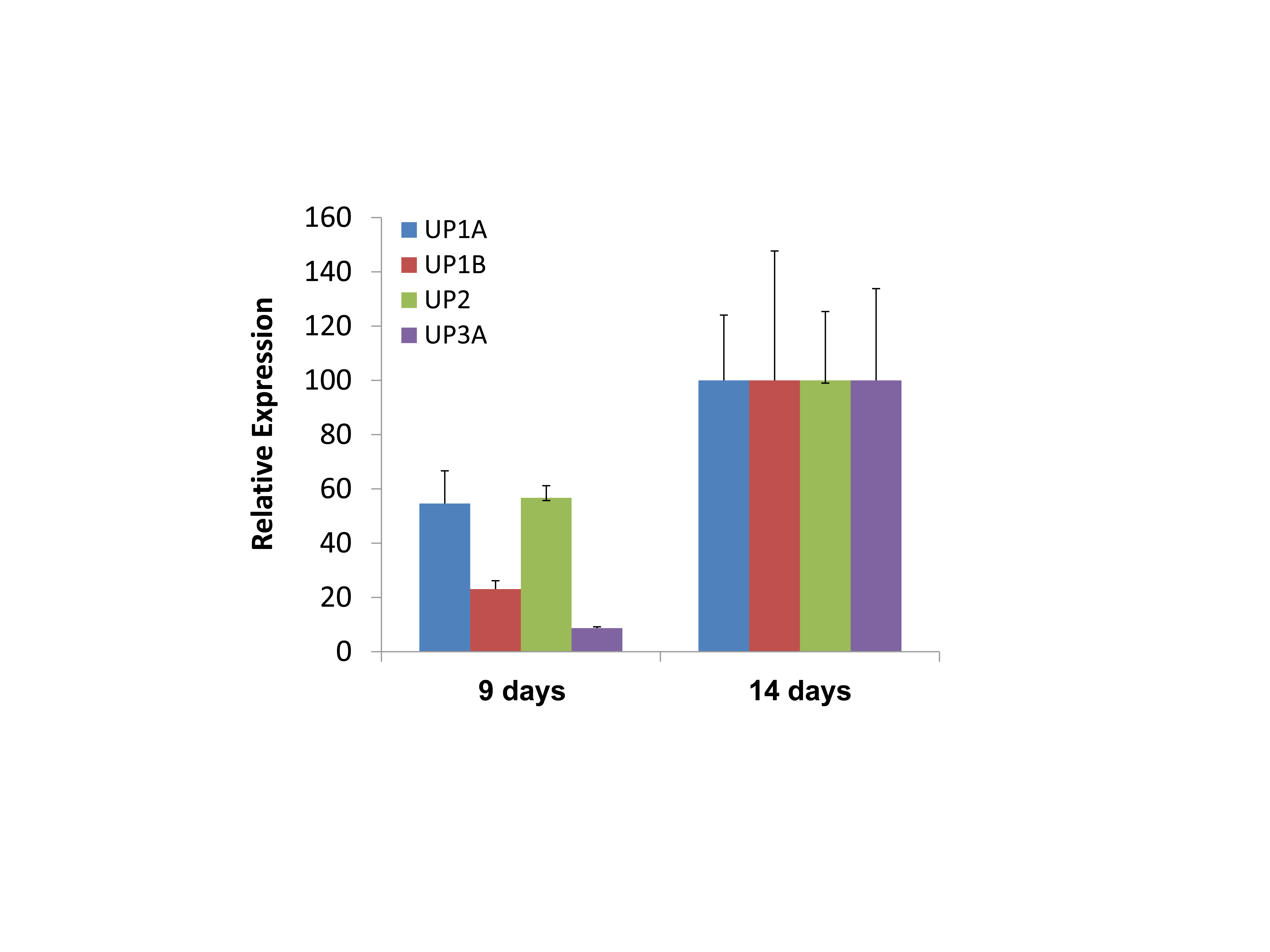

Supplement: Figure S1 — RA stimulation of ESCs further induces uroplakin expression from 9 to 14 days of culture. Real time RT-PCR analysis of UP expression by ESCs cultured on collagen matrices in the presence (+RA) or absence (C) of 10 µM RA from 9 to 14 d of culture. Levels normalized to GAPDH expression. Mean ± SD per data point. (0.44 MB TIF) [file pone.0011513.s001.tif]

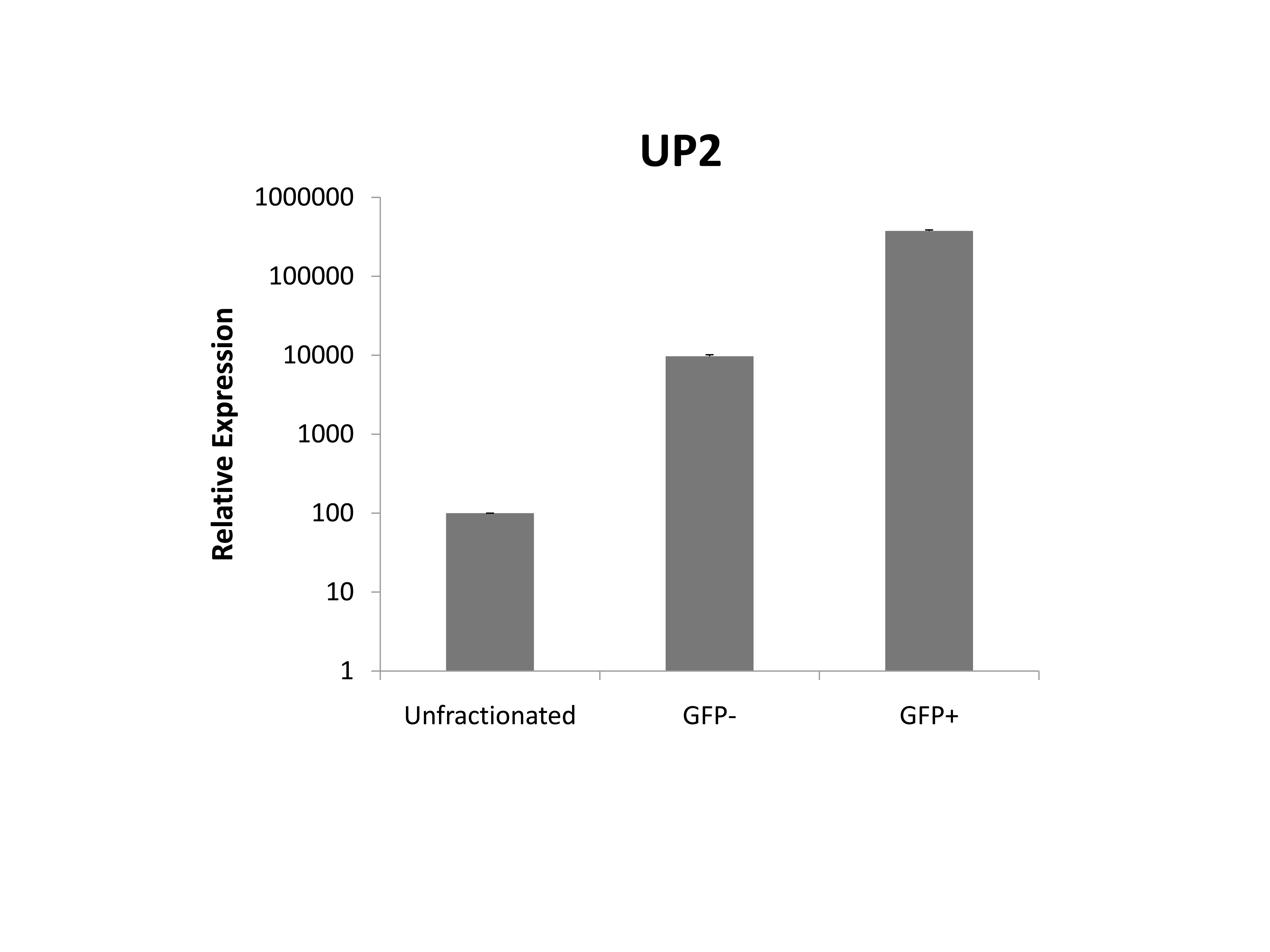

Supplement: Figure S2 — Endogenous uroplakin 2 expression of FACS-sorted UP2-GFP ESC lines in response to RA stimulation. Real-time RT-PCR analysis of endogenous UP2 expression in UP2-GFP ESC lines cultured for 14 d in the presence of RA. Samples consisted of either unfractionated controls, GFP positive and negative fractions. Levels normalized to GAPDH expression. Mean ± SD per data point. (0.42 MB TIF) [file pone.0011513.s002.tif]

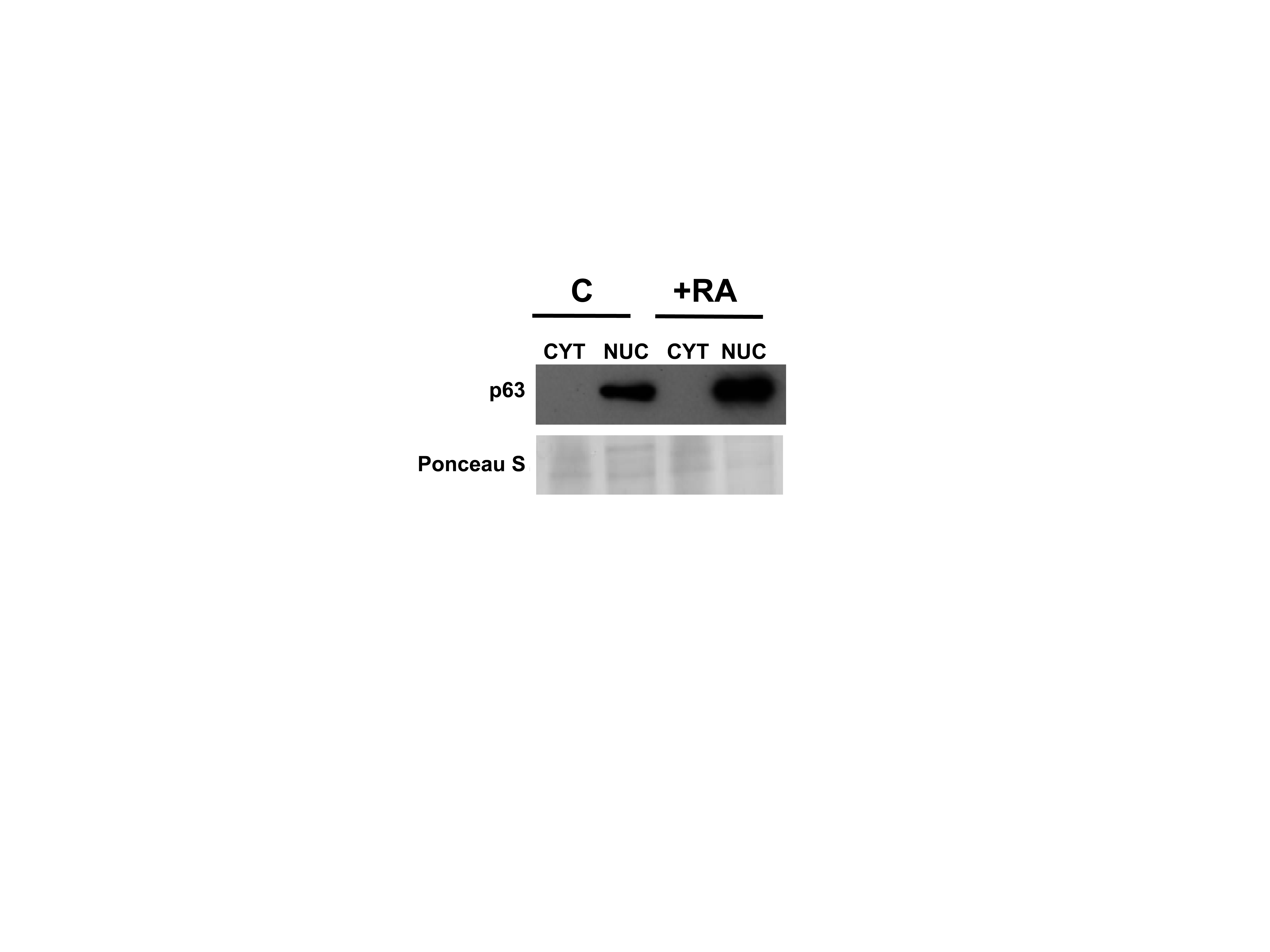

Supplement: Figure S3 — Enrichment of p63 in nuclear fractions of RA-treated ESCs. Immunoblot analysis of nuclear (NUC) and cytoplasmic (CYT) fractions demonstrating enrichment of p63 in nuclear extracts of wild type (WT) RA-treated ESCs following 9 d of cultivation. (0.49 MB TIF) [file pone.0011513.s003.tif]

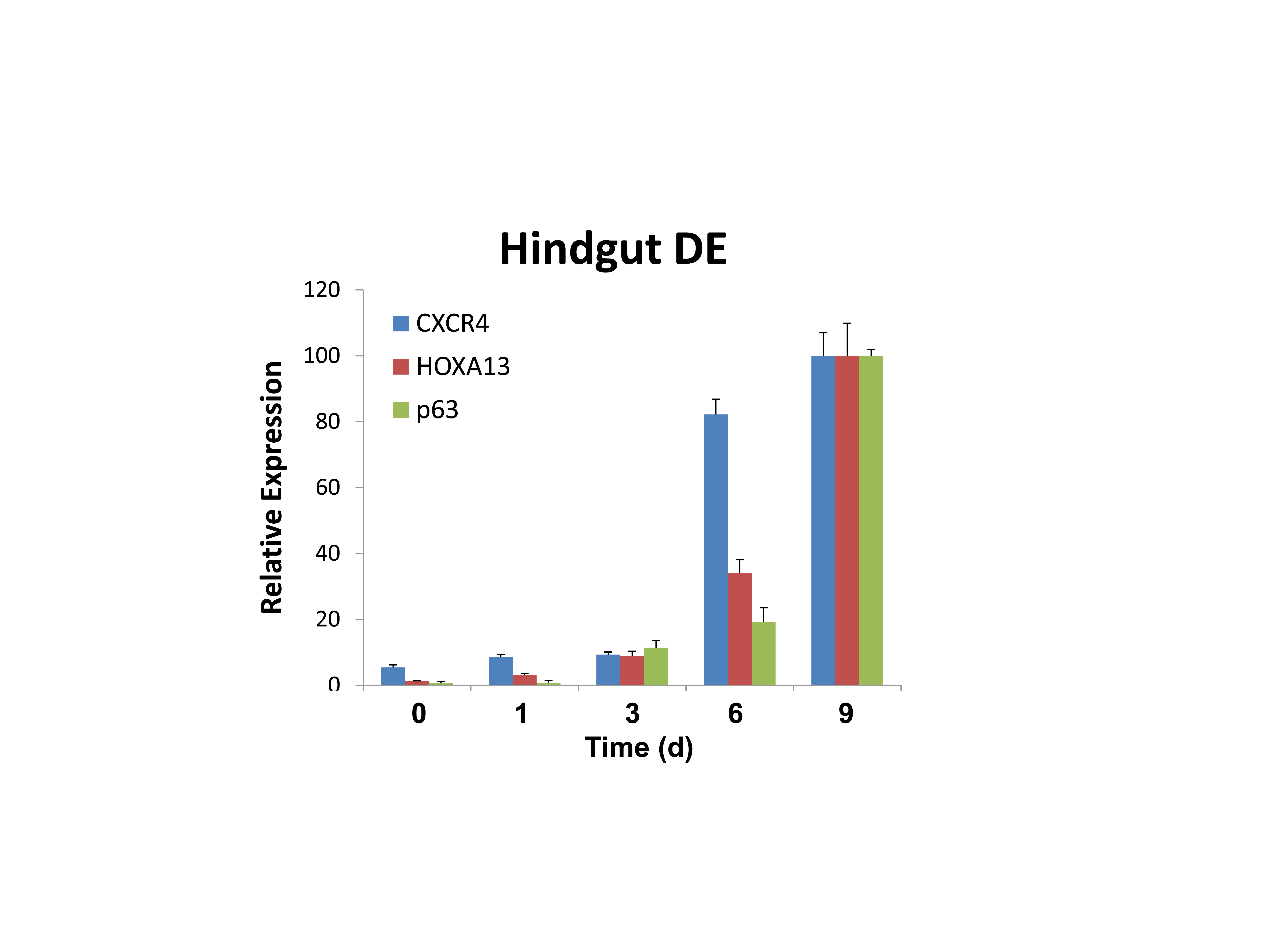

Supplement: Figure S4 — RA stimulation of ESCs promotes upregulation of hindgut definitive endoderm markers in a time dependent manner. Real time RT-PCR analysis of definitive hindgut endoderm markers in response to RA stimulation (10 µM) over 9 d of cultivation. Undifferentiated and spontaneously differentiating controls (C) were analyzed in parallel. Levels normalized to GAPDH expression. Mean ± SD per data point. (0.44 MB TIF) [file pone.0011513.s004.tif]

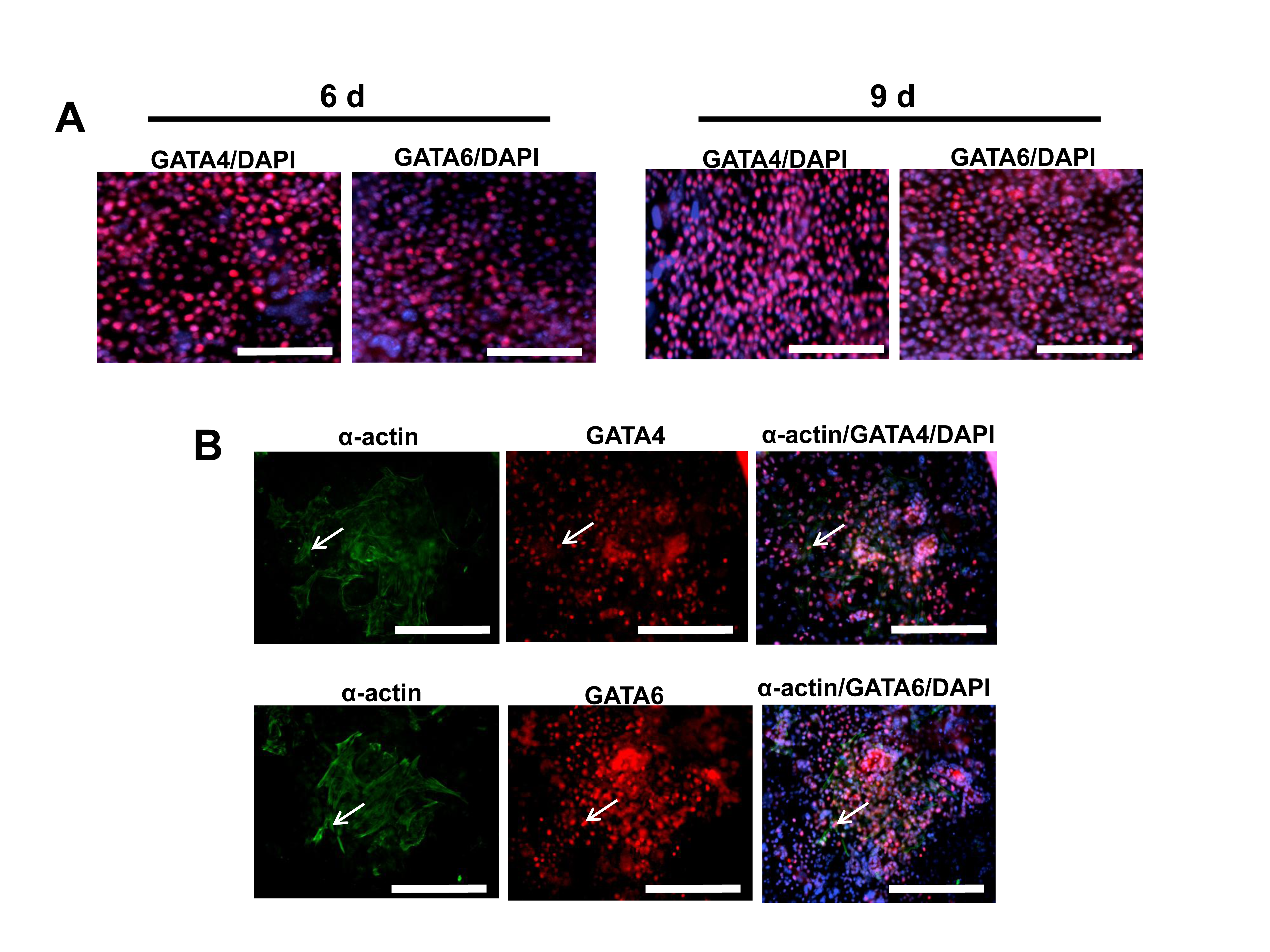

Supplement: Figure S5 — RA stimulation of ESCs promotes temporal expression of nuclear GATA4/6 in various subpopulations including SMC phenotypes. [A] Nuclear GATA4/6 expression in RA-treated ESCs following 6 and 9 d of culture. Scale bar = 60 µm. [B] Photomicrographs of RA-treated ESCs co-stained for α-actin (green, FITC) and nuclear GATA4/6 (red, Cy3) (denoted by white arrows) following 14 d of culture. Scale bar = 500 µm. For both [A, B], images were merged with DAPI nuclear counterstain (blue). (9.41 MB TIF) [file pone.0011513.s005.tif]
